# Progress in Research on Stem Cells in Neonatal Refractory Diseases

**DOI:** 10.3390/jpm13081281

**Published:** 2023-08-21

**Authors:** Fangjun Huang, Yang He, Meng Zhang, Keren Luo, Jiawen Li, Jiali Li, Xinyu Zhang, Xiaoyan Dong, Jun Tang

**Affiliations:** 1 Department of Neonatology, West China Second Hospital, Sichuan University, Chengdu 610041, China; 2 Key Laboratory of Birth Defects and Related Diseases of Women and Children (Sichuan University), Ministry of Education, Chengdu 610041, China

**Keywords:** stem cells, hypoxia–ischemia, cerebral intraventricular hemorrhage, bronchopulmonary dysplasia, necrotizing enterocolitis, retinopathy of prematurity

## Abstract

With the development and progress of medical technology, the survival rate of premature and low-birth-weight infants has increased, as has the incidence of a variety of neonatal diseases, such as hypoxic–ischemic encephalopathy, intraventricular hemorrhage, bronchopulmonary dysplasia, necrotizing enterocolitis, and retinopathy of prematurity. These diseases cause severe health conditions with poor prognoses, and existing control methods are ineffective for such diseases. Stem cells are a special type of cells with self-renewal and differentiation potential, and their mechanisms mainly include anti-inflammatory and anti-apoptotic properties, reducing oxidative stress, and boosting regeneration. Their paracrine effects can affect the microenvironment in which they survive, thereby affecting the biological characteristics of other cells. Due to their unique abilities, stem cells have been used in treating various diseases. Therefore, stem cell therapy may open up the possibility of treating such neonatal diseases. This review summarizes the research progress on stem cells and exosomes derived from stem cells in neonatal refractory diseases to provide new insights for most researchers and clinicians regarding future treatments. In addition, the current challenges and perspectives in stem cell therapy are discussed.

## 1. Introduction

In 1960, American neonatologist Alexander Schaffer first proposed the concept of neonatology. After more than 60 years of pediatric medical development, the survival rate of premature and low-birth-weight infants has improved. With the extensive use of ultrasound, CT, MRI, ventilator, central venous catheterization, broad-spectrum antibiotics, and other diagnostic techniques, an increasing number of neonatal illnesses have been identified, such as hypoxic–ischemic encephalopathy (HIE), bronchopulmonary dysplasia (BPD), intraventricular hemorrhage (IVH), necrotizing enterocolitis (NEC), and retinopathy of prematurity (ROP) [1,2,3,4,5,6,7,8]. Patients with these diseases often have severe conditions, long disease courses, high costs, and poor prognoses. Under conventional treatment, clinicians focus on whether to open a new track.

**Figure 1:**
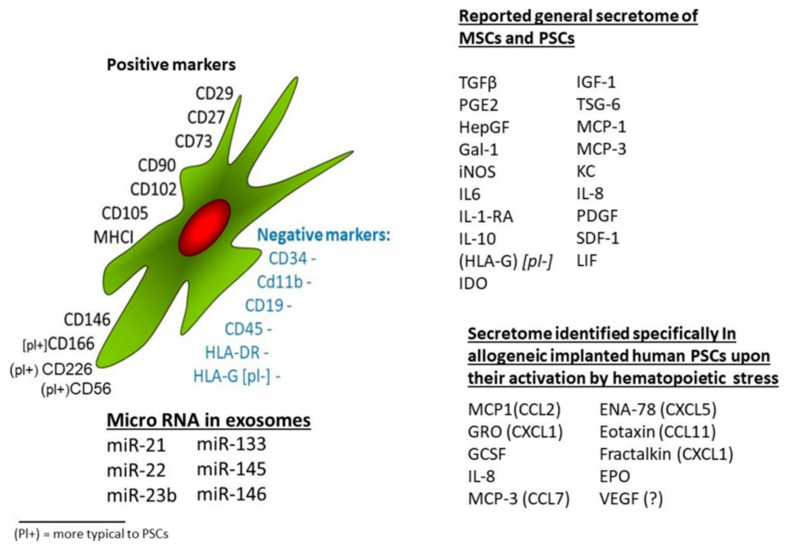
Stem cells from different sources have a wide range of common characteristics in terms of cell surface markers, growth factor secretion profiles, and cytokines [9].

Stem cells (SCs) are a special type of cells with the potential to self-renew and differentiate. They exist in an undifferentiated or poorly differentiated state, and their paracrine effects can affect the microenvironment in which they live, thereby affecting the biological characteristics of other cells. The markers and secretions of mesenchymal stem cells (MSCs), exosome content, and secretome of human pluripotent SCs (hPSCs) are shown in Figure 1 [9,10]. In recent years, MSCs have become the main candidate cells for SC treatment because of their wide range of sources, such as the myocardium, blood vessels, nerves, urine, bone marrow, fat, amniotic fluid, and umbilical cord blood. The unique biological characteristics of MSCs—pluripotency, self-renewal and paracrine activity, and particularly the ability of nutrient regulation, immunomodulation, and migration to injured sites [11,12,13]—are well suited for cell-based therapy, such as the replacement of dead or defective cells and delivery of genes or drugs from damaged tissue to injured sites as delivery agents [14,15]. Therefore, MSCs have great potential for use in degenerative diseases, immune diseases, hematologic diseases, organ transplantation, and regenerative medicine [16,17,18,19,20,21,22,23].

Many countries have registered dozens of clinical trials of SCs for neonatal diseases, providing possibilities for early application [24]. Neonatal medicine endeavored to achieve high-quality survival by preventing complications and improving neurological development in vulnerable groups [25]. This review aims to summarize recent research progress on SCs and exosomes derived from SCs in neonatal refractory diseases. In addition, this review discusses the current challenges and perspectives of stem cell therapy.

## 2. Types of SCs

According to their differentiation potential and source, the types of SCs commonly used in neonatal refractory diseases are highlighted in the following sections: (1) Bone marrow mesenchymal stem cells (BM-MSCs), derived from the mesoderm, can self-renew, proliferate, differentiate, and differentiate into osteoblasts, cartilage, fat, muscle, nerve, and other tissue cells through induction. The paracrine function of BM-MSCs is as important as that of SCs. They can secrete many types of cytokines from the bone marrow, such as blood and bone growth factors [26]. Additionally, BM-MSCs, with the advantages of low immunogenicity, easy access to materials, comprehensive sources, no ethical issues, and easy-to-industrialize preparation [27], were commonly used to treat neonatal diseases. (2) Umbilical cord blood mesenchymal stem cells (UCB-MSCs), derived from umbilical cord blood progenitor cells, can differentiate into cells derived from the mesoderm such as fat cells, osteoblasts, muscle cells, hepatocytes from the endoderm, and nerve cells from the ectoderm [28]. Several studies have found that neurotrophic and anti-apoptotic factors secreted by UCB-MSCs have an anti-inflammatory ability, induce neurogenesis and vasculogenesis, accelerate nerve recovery, and improve neurobehavioral outcomes [29,30]. Studies have also found that UCB-MSCs increase angiogenesis and vascular stability in stroke patients treated with angiopoietin/tie [31]. Therefore, UCB-MSC transplantation not only has the advantages of rich sources, easy collection, preservation, non-invasiveness, no ethical issues, and less antigenicity but also secretes a variety of cytokines to promote angiogenesis, neuronal differentiation, nerve repair, and protection and plays an important role in neurological diseases in newborns. (3) Human amniotic fluid SCs (hAFSCs) are a group of embryonic and adult SCs. They have a multi-directional differentiation potential; they can differentiate into endodermal, mesodermal, and ectodermal cells and can be expanded in vitro without trophoblastic cells. Amplification does not involve tumorigenesis [32] and is safer for treating neonatal diseases. The mechanism of action of AFSCs is unclear; however, they are more readily available and cultured than MSCs from other sources to achieve clinical transformation [32,33].

There are still some other common SCs, but they may be less common in neonatal disease research applications; examples include the following: (1) In vitro and in vivo studies have shown that human adipose-derived MSCs (hADSCs) are pluripotent and can differentiate into mesoderm-derived cells, including fat cells, chondrocytes, and osteoblasts [34]. hADSCs enter the surrounding environment through secretion or production of a range of cytokines, growth factors, nucleic acids (miRNAs), and other macromolecules through microvesicles, altering histobiology, stimulating tissue-resident SCs, altering immune cell activity, and mediating treatment outcomes [35]. Their advantages include abundant sources, easy tissue collection and cell isolation, and therapeutic potential [34]. hADSCs have been widely used in adult clinical and experimental research [36]. However, they are currently used less commonly in neonatal disease research. (2) Placenta-derived mesenchymal stem cells (PMSCs) can be classified as intermediates between BM-MSCs and embryonic SCs (ESCs) [37]. Compared to BM-MSCs, they have multipotent and self-renewing properties without the disadvantages associated with using ESCs [37]. PMSCs treat various diseases, including cancer, neurological disorders, bone diseases, and cardiovascular diseases. Their advantages are that they are abundant, easy to obtain, and stored after birth [38]. There were no mortality problems; source cells were less affected by age and environmental factors [37]. (3) In addition, some adult SCs, such as human-induced neural SCs, can be used to promote the recovery of function in spinal cord injury models [39], epilepsy, mental disorders, intellectual disabilities, and other brain diseases [40]. Muscle SCs offer hope to the muscular dystrophy patient population [41]. Bone-marrow-derived SCs (BMSCs) play important roles in bone regeneration [42]. Although iPSCs are not abundant in the neonatal population, as an important stem cell population, their strong multi-directional differentiation ability remains an important technology in cell therapy research. iPSCs artificially induce stemness by “resetting” adult differentiated cells (e.g., differentiation into hematopoietic SCs and progenitor cells) [43]; they are used in the construction of in vitro models of diseases, such as type III Gaucher disease and Down syndrome/trisomy 21 [44]; based on regenerative medicine research, they are used in the construction of autologous tissue heart organoids [45], reduce the lack of natural pluripotent SCs, and have low ethical risk and high carcinogenic risk. The types of SCs are abundant. Currently, only a few types of SCs are used in neonatal disease research, and further trials are needed.

## 3. Mechanisms of SC Therapy

**Figure 2:**
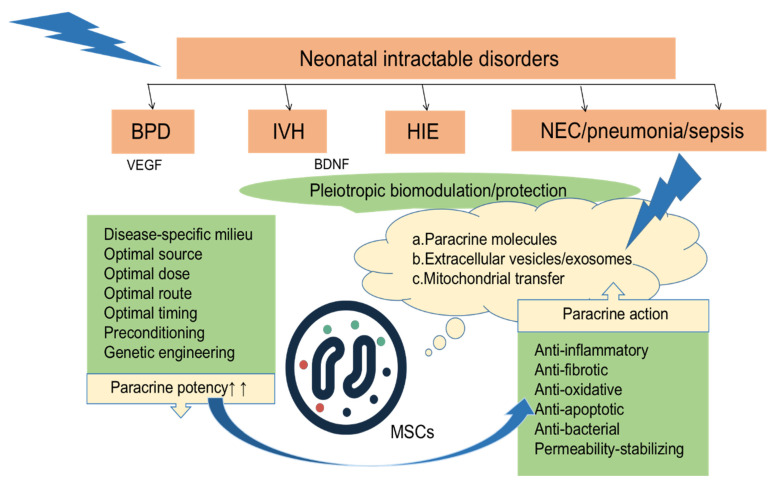
After local or systemic transplantation of MSCs to various tissue injury sites, they exert paracrine effects such as anti-inflammatory, anti-fibrotic, antioxidant, anti-apoptotic, antibacterial, and stable permeability effects by secreting a variety of factors [50].

The mechanisms of SC therapy include anti-inflammatory and anti-apoptotic effects, oxidative stress reduction, and boosting regeneration [46]. The mechanism by which SCs are implanted at high levels in multiple tissues when the body is damaged or inflamed is not fully understood [47]. Some researchers have suggested that this may be related to the specific expression of receptors or ligands in the injured tissue to promote the directional transport, adhesion, and infiltration of SCs to the injured site [48]; repair of the injury occurs not entirely through the transport of exogenous cells to replace the damaged cells, but also through the release of nutritional factors to improve endogenous repair [49,50]. The paracrine mechanisms of polytropic biological regulation and the protection of MSCs against refractory neonatal diseases such as BPD, IVH, and HIE are shown in Figure 2 [50].

SCs are a special type of cells with self-renewal and differentiation potential, with a wide range of sources, such as the myocardium, blood, fat, and bone marrow. They exist in an undifferentiated or poorly differentiated state. They can differentiate into various tissue cells under certain conditions, such as cardiomyocytes, muscle cells, intestinal cells, and nerve cells, to promote tissue regeneration. However, their directional differentiation ability is poor, the survival rate is low, and the risk of tumor formation is high. Therefore, current treatment with SCs does not exclusively replace damaged cells by transporting exogenous cells but also improves endogenous repair by releasing nutritional factors [49]. In most cases, SC therapy is indirect, not by proliferation or differentiation at the injury site, but by a paracrine mechanism that secretes an extracellular vesicle containing various proteins, lipids, and nucleic acids. Depending on their size and biological pathways, they can be divided into three types: exosomes, approximately 30–100 nm, derived from vesicles in polycytes fused with the plasma membrane; microvesicles, approximately 100–1000 nm, formed directly from a detachment of the plasma membrane; and apoptotic bodies, >1000 nm, from apoptosis. Such extracellular vesicles protect nerves, fight inflammation, fibrosis, oxidation, and apoptosis, and reduce permeability [50,51,52,53,54] by releasing neuroprotective, neurogenic, and anti-inflammatory factors [9,55], such as brain-derived neurotrophic factor (BDNF) [56]. For example, BDNF can protect against brain injury induced by severe IVH by reducing the risk of inflammatory factors, astrocyte hyperplasia, and hydrocephalus after hemorrhage and improving sensory and behavioral abilities [52]. Other studies suggest that MSCs transfected with the BDNF gene can attenuate OGD-induced cytotoxicity, oxidative stress, and cell death in vitro by more than five times the capacity of original MSCs, and that only these cells show significant attenuation of short-term brain injury score, long-term progression of cerebral infarction, increased apoptosis, astrocyte proliferation, and inflammatory response induced by severe HIE [57]. Previous studies have shown that SCs are less abundant in the brain, intravenously and intraperitoneally [58]. However, the main therapeutic mechanism of SCs is the production of a variety of cytokines for multi-directional regulation to achieve cascade-like signaling effects so that even a small number of SCs can act as therapeutic agents through the blood–brain barrier [57,59,60,61,62,63,64,65,66,67,68,69]. SCs have similar effects on other lesions, which can be explained by their different mechanisms of action in various diseases.

## 4. Application of SCs in Refractory Diseases of Newborns

### 4.1. Hypoxic–Ischemic Encephalopathy

HIE refers to fetal and neonatal brain injury caused by various factors such as perinatal hypoxia and cerebral blood flow reduction or suspension. Currently, the treatment of HIE mainly involves therapeutic hypothermia [70]. However, mild hypothermia is only effective within 6 h of the onset of severe HIE, and more than half of children die or have severe neurological problems after active treatment [71]. Therefore, there is an urgent need to find new treatments that can prolong the treatment window and improve the poor prognosis of HIE.

Brain injury can be classified into five processes: energy exhaustion, inflammation, cellular excitotoxicity, oxidative stress, and apoptosis [46]. Hypothermia does not significantly reduce brain damage primarily by reducing energy depletion and increasing intracellular calcium ion levels [72]. In a study of HIE in term newborns and stroke in adults, MSCs played a major role in boosting the proliferation and differentiation of nerve cells and regulating or suppressing the local immune response of microglia and T lymphocytes [73]. In addition, MSCs and exosomes secreted by hPSCs may be a promising treatment for neuronal injury by interacting with parenchymal cells in the brain, resulting in reduced expression of axonal inhibitors and increased production of neuroprotective factors, thereby affecting axonal growth and promoting the restoration of normal nerve function [74].

**Figure 3:**
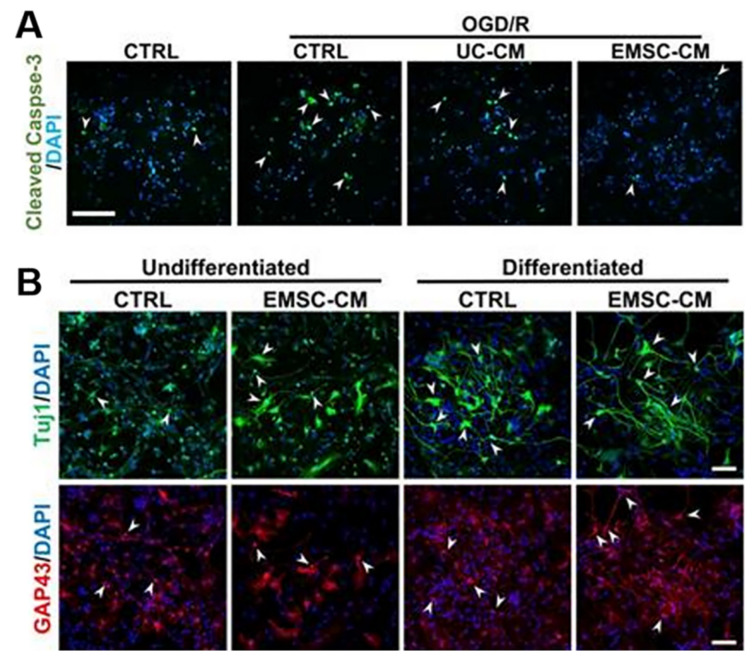
(**A**) Primary cultures of rat cortical neurons were exposed to OGD/R in the presence or absence of CM derived from hUC-MSCs or hPSC-EMSCs. The results showed a significant reduction in the number of apoptotic cells after OGD/R attack following incubation with CM, with fewer apoptotic cells observed in the EMSC-CM-treated group than in the control group. Arrowheads indicate cleaved caspase-3-positive cells. (**B**) Representative immunofluorescence staining for Tuj1 and GAP43 showed that higher levels of neuronal phenotypic differentiation were observed in CM-treated rat neuronal precursor cells [64]. Arrowheads indicate Tuj1 or GAP43 positive cells. Scale bar, 100 µm.

**Figure 4:**
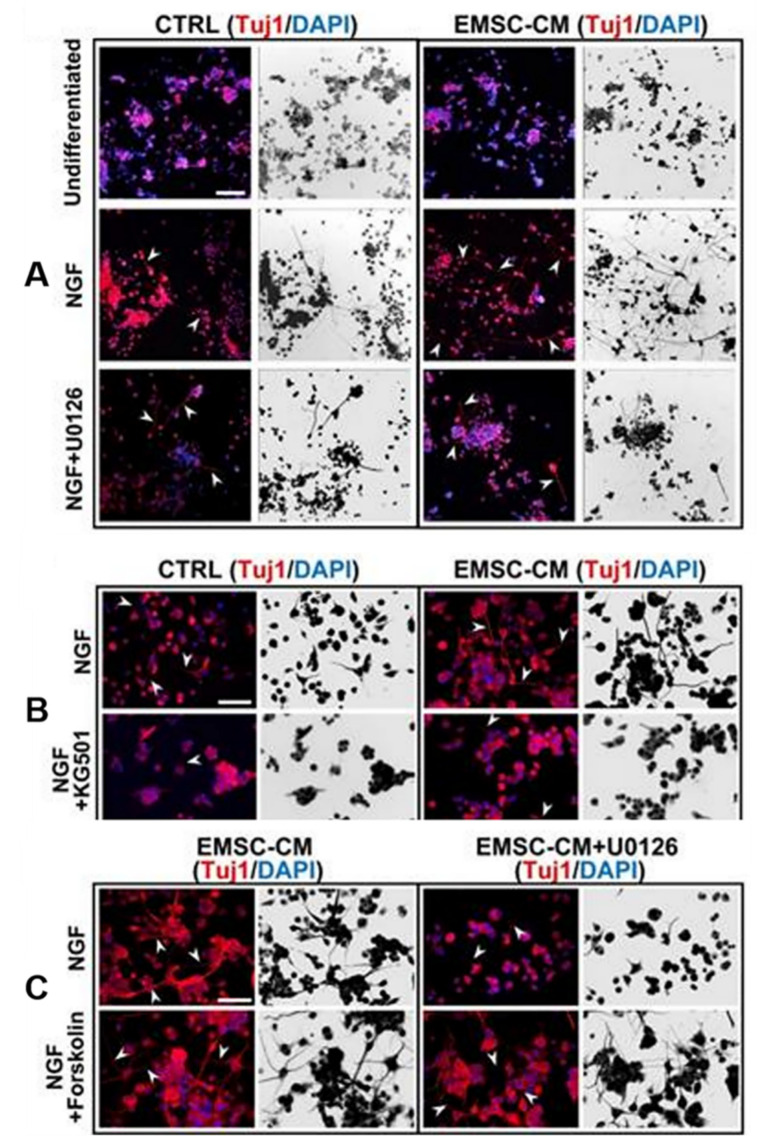
Conditioned medium from human pluripotent stem-cell-derived ectomesenchymal stromal cells promotes neurogenesis and neuritogenesis via the ERK/CREB signaling pathway. (**A**,**B**) Arrowheads indicate cells with neurites. U0126 and CREB inhibitor KG501 completely abolished CM-enhanced neurogenesis in PC-12 cells. (**C**) The activation of CREB by forskolin promoted neurogenesis. U0126 did not attenuate this effect, suggesting that CREB is downstream of ERK [64]. Arrowheads indicate PC-12 cells with neurites. Scale bar, 100 µm.

**Figure 5:**
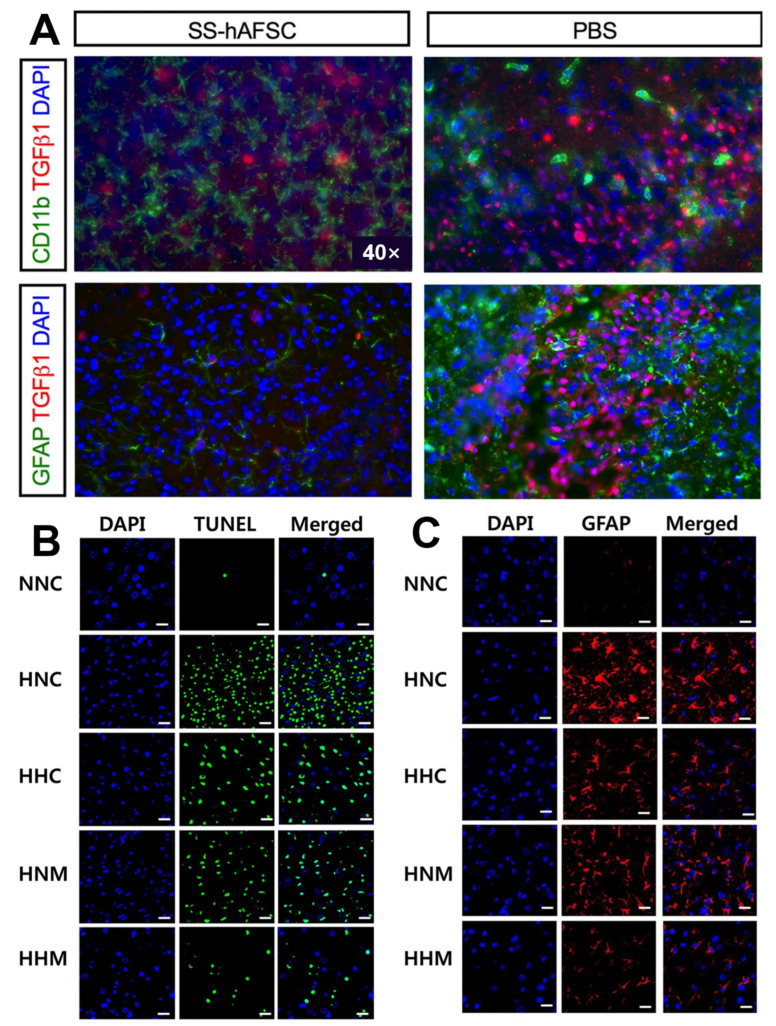
(**A**) The co-immunostaining in the hippocampus of HI mice transplanted with SS-hAFSCs and PBS only, showing reduced levels of TGFβ1 (red staining), CD11b (green staining), and GFAP (green staining) in SS-hAFSC-transplanted mice [79]. (**B**) Representative immunofluorescence micrographs show that hypothermia treatment significantly ameliorated HIE-induced cell death, and combined hypothermia and delayed MSC treatment provided better attenuation than single hypothermia treatment [85]. Scale bar, 25 μm. (**C**) Hypothermia combined with delayed MSC treatment was superior to subcooling alone or MSCs alone in the treatment group. Ambient temperature inside the chamber was 34.0 °C for normothermia (HNC, HNM) and 31.0 °C for hypothermia (HHC, HHM) [85]. Scale bar, 25 μm.

There are several sources of SCs. Selecting the most suitable SCs for clinical applications based on safety, functional effects, or similar features is very important. BM-MSC implantation can reduce lesion volume and inflammatory cells and promote cell differentiation into neurons and oligodendrocytes, thereby improving neonatal HIE brain damage [75]. Compared to BM-MSCs, hADSCs are more readily available, have fewer ethical issues, and can produce more vascular endothelial growth factor (VEGF) and hepatocyte growth factor (HGF) after 1–2 days of culture, with a difference of less than 10% in the cytokine spectrum between the two [76]. UCB-MSCs can inhibit neuronal death, reduce apoptosis and injury volume after HIE injury, and inhibit the secretion of inflammatory factors such as TNF-α and IL-1β [77]. In addition, a recent study suggested that compared to human umbilical cord mesenchymal stem cells (UC-MSCs), extracorporeal mesenchymal stromal cells derived from hPSCs have greater differentiation potential, can be expanded indefinitely, and use the ERK/CREB pathway to inhibit inflammation, reduce apoptosis, protect nerves, promote nerve regeneration, and improve motor function in HIE mouse models through unique bioactive factors (such as nerve growth factor and platelet-derived growth factor-AA) [64] (Figure 3 and Figure 4). A study found that hAFSCs in rodent models of HIE and periventricular leukomalacia not only inhibited neuronal inflammation and repaired neuronal cells [78], but also improved sensorimotor impairment, reduced brain injury volume, and induced gene expression of neurotrophins and chemokines in the chronic phase [67]. Moreover, it has been demonstrated that the neuroprotective effect of spindle-shaped AFSCs in hypoxic–ischemic mice may be achieved by inhibiting the endogenous inhibition of TGFβ1 [79] (Figure 5A). Therefore, SCs from different sources have different disease prevention and treatment mechanisms, and further research is needed to explore which stem cells provide the best rapidity. 

Different administration times, dosages, and methods in clinical applications also influence therapeutic effects. Studies have shown that delayed administration (1 week after HIE) of large doses of BM-MSCs (7.5 × 10^5^–1.0 × 10^6^ cells) can restore neurons projected from the striatum and improve motor function [80]. Other studies have suggested that delaying the administration of double high doses of hADSCs (PN14, PN16, ih, 1.07 × 10^6^, qd) in HIE rats significantly increases the absolute number of dorsal striatum dopamine and cyclic adenylate-regulated phosphoprotein-positive spinel neurons to normal undamaged levels [73]. Donega et al. found that MSCs could be found at the site of injury 10 days after HIE, but not at 17 days, proving that the SC treatment window is at least 10 days, significantly extending the time window for hypothermia and bringing more hope for children with HIE [81]. In conclusion, high-dose SCs positively affect the HIE model, and the therapeutic window is longer than that of hypothermia treatment; therefore, they are more accessible in the clinic. Local intracerebroventricular injection (IC) has been shown to have better paracrine and therapeutic efficacy than intravenous injection (IV) [82], and some studies have reported pulmonary embolism caused by intravenous or intra-arterial injection of MSCs [83,84]. Hattori et al. suggested that the intraperitoneal injection (IP) of umbilical cord blood stem cells (UCB-SCs) within 24 h could induce anti-apoptotic and antioxidant effects and reduce the number of activated microglia [58]. However, these changes are temporary, and no chronic long-term changes (such as morphological or functional changes) have been observed, and repeated administration or combination therapy may be required to achieve sustained protective effects [43]. Vanessa et al. also found that single intranasal doses of MSCs on days 3, 10, and 17 after HIE reached the lesion site within 24 h, 3 days, and 10 days after injury, reducing brain damage, improving sensorimotor and cognitive function, and leading to lasting (9 weeks) improved tissue outcomes [81]. Regardless of the injection method, the SC treatment had a certain effect. IC treatment is effective and does not easily lead to pulmonary embolism or other complications; however, it is more traumatic in newborns and has a narrow application range. Therefore, it is necessary to develop cost-effective injection methods. Regarding treatment options, concurrent or delayed injection of MSCs can synergistically improve severe HIE compared to hypothermia alone [82,85]. Hypothermia broadens the therapeutic time window for MSC transplantation in severe neonatal hypoxic–ischemic encephalopathy [85] (Figure 5B,C). A meta-analysis involving three studies [86] concluded that hypothermia combined with MSCs reduced astrocyte proliferation, but there were insufficient preclinical data to demonstrate that hypothermia combined with MSC is superior to hypothermia alone, and more preclinical trials and further clinical trials are needed. Basic research for HIE based on stem cell therapy is presented in Table 1.

In a one-arm clinical study in Japan, patients used different doses of autologous hUCB-SCs at 12–24, 36–48, and 60–72 h after the diagnosis of HIE and were followed up until 18 months after birth. No patients died, four had no significant sequelae, and two developed cerebral palsy [87]. It has been proven that if enough hUCB-SCs can be used in the clinic, they are safe and feasible and reduce the risk of immunogenicity and ethical concerns. However, as this was a small one-arm trial, the data were not sufficiently representative, and more studies are needed to prove the feasibility of SC treatment. Another clinical study showed that children with HIE who received human UCB-SCs combined with hypothermia had a slightly better survival rate and a Bayley III score (cognitive, language, and motor development) ≥ 85 (74% vs. 41%) compared to those who received hypothermia alone [88]. MSCs are expected to become the best treatment for HIE due to their advantages of relatively easy access, low immunogenicity, and promotion of nerve regeneration. To date, only nine trials have been registered at www.clinicaltrials.gov (accessed on 20 July 2023): one has been completed (NCT02287077), one has completed recruitment (NCT02881970), two are active but have not completed recruitment (NCT04063215, NCT04261335), one has not completed recruitment (NCT05514340), three are unknown (NCT01962233, NCT02854579), and one has been withdrawn (NCT02434965). Existing studies suggest that SC therapy is feasible and safe; however, the number of enrolled patients is small, and more phase 3 clinical trials are needed to obtain more data, such as long-term prognosis and neurological evaluation. There is great controversy about stem cells in terms of plasticity, for example, the low conversion rate of stem cells from non-nervous system sources to neural cells. The in vitro regulation of stem cell migration, proliferation, and differentiation needs to be further solved. Moreover, the mechanism of stem cell transplantation in the treatment of HIE is not completely clear, mainly including the cell replacement effect, immunomodulation effect, and paracrine factor effect.

### 4.2. Intraventricular Hemorrhage

The incidence of IVH in infants with extremely low and very low birth weight is high [89], and the mediated white matter damage can lead to hydrocephalus, cerebral palsy, mental retardation, and other complications [90]. The main pathogenesis may include IVH-induced cerebral inflammation, oxidative stress in the white matter, and myelin deficiency [91]. Currently, supportive treatment is available, but no other prevention or treatment is available. Therefore, developing a treatment that can reach the injured central nervous system more quickly is necessary to reduce inflammation and astrocyte hyperplasia.

**Figure 6:**
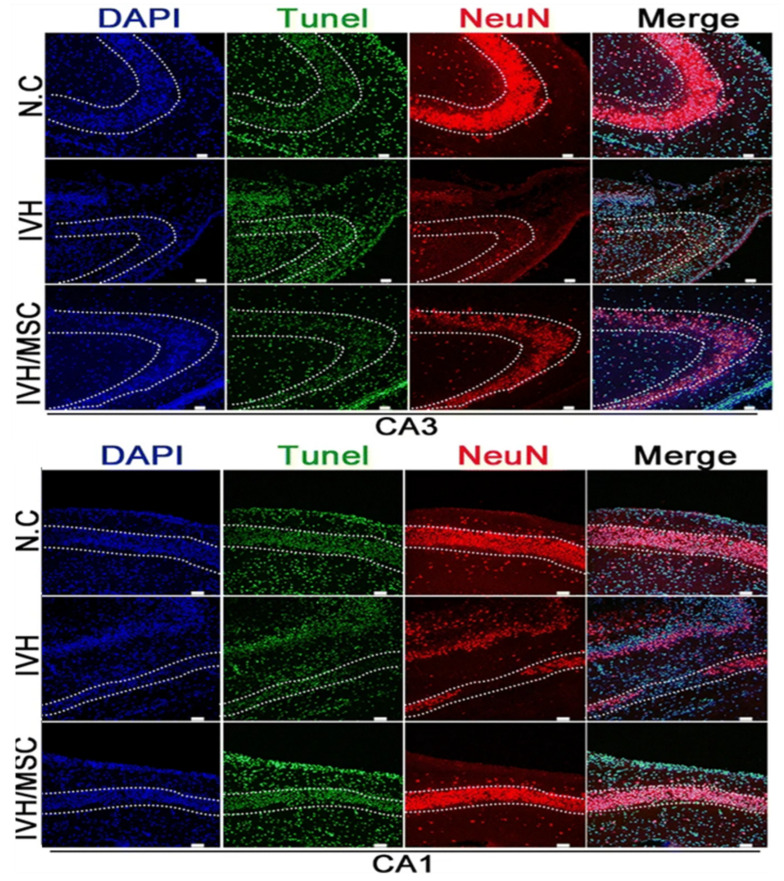
Images of TUNEL staining and immunohistochemical staining. DNA stained with DAPI (blue). Nuclei of TUNEL-positive cells stained green. Neuronal marker NeuN stained (red). There was notable TUNEL staining in the CA3-CA1 region of the hippocampus of IVH without hUCB-MSC treatment, whereas the TUNEL fluorescence intensity in the hippocampus of hUCB-MSC-treated rats after IVH injury was only about one-fourth of that in the IVH model [93]. Scale bar, 50 μm.

**Figure 7:**
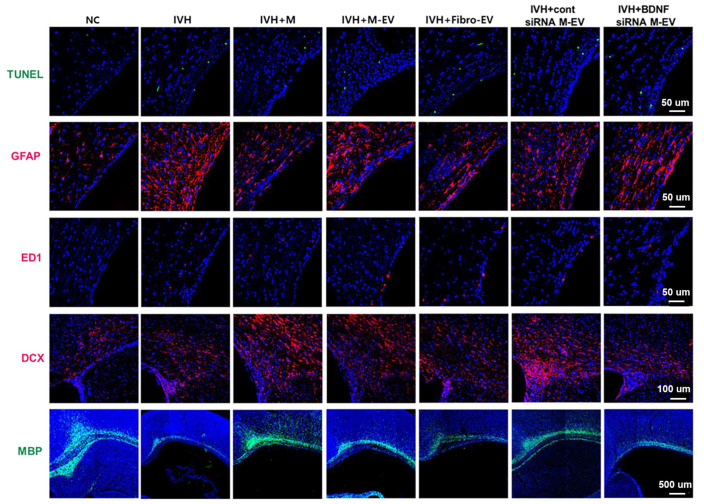
Representative immunofluorescence micrographs of periventricular areas with TUNEL (green), glial fibrillary acidic protein (GFAP) (red), ED1 (red), double corticosteroid (DCX) (red), and myelin basic protein (MBP) (green) staining. BDNF knockdown in extracellular vesicles eliminates the therapeutic effect of MSCs in improving brain myelin formation and attenuating cell death and reactive gliosis after severe IVH [94].

In IVH, hUCB-MSCs reduce TNF-α, improve synaptic dysfunction in the thalamic cortex [92], promote the survival of hippocampal neurons, reduce the loss of hippocampal neurons, reduce hippocampal synaptic circuit damage, and restore behavioral function [93] (Figure 6). Owing to the unique application value of extracellular vesicles in the brain, their content-specific effects and comparison with MSCs have also been the focus of research. Exosomes derived from hUCB-MSCs contain BDNF and have effects similar to those of hUCB-MSCs in the treatment of IVH, such as reducing neuronal cell and TUNEL-positive apoptotic cell death; reducing the inflammatory response, oxidative stress, and brain damage; and improving sensorimotor function after IVH [94] (Figure 7). Therefore, under similar conditions, treatment of extracellular vesicles in the brain may have more clinical significance.

**Figure 8:**
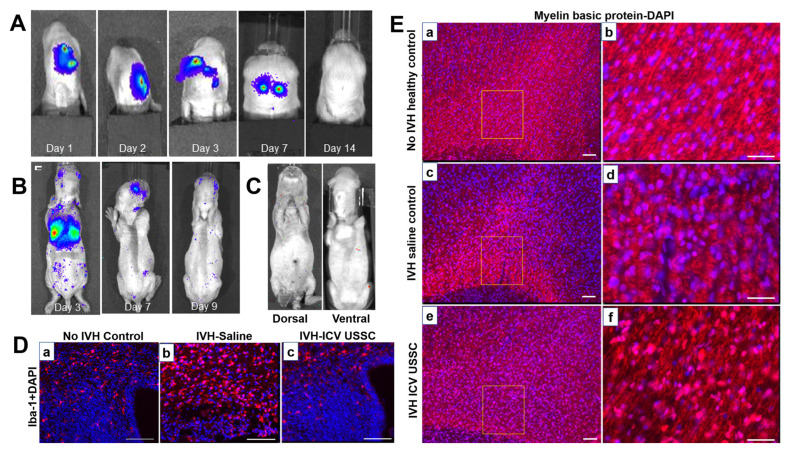
(**A**) Bioluminescence live images (BLIs) of unrestricted somatic stem cell (USSC) engraftment by intracerebroventricular (ICV) and intravenous (IV) delivery in IVH pups. (**B**,**C**) BLI signals in dissected whole brain and coronal brain sections from IVH pups injected with USSC on day 3. Scattered luminescent signals in the brain indicate further migration of USSCs. (**D**) Representative immunofluorescence images of microglia labeled with Iba-1-specific antibodies at postnatal day 3. Specific Iba-1 antibodies in the IVH group were more responsive to immunization of microglia compared with no-IVH control in germinal matrix and corona radiata (**a**,**b**). Also, ICV-administered USSC pups showed reduced immunoreactivity to microglia compared to the IVH group (**c**). Scale bar, 100 µm. (**E**) Morphological changes of myelin fibers in corona radiata were assessed by immunolabeling of myelin basic protein (MBP) at day 14. ICV USSC administration preserves myelin after IVH in premature rabbit pups [97]. (**a**,**b**): Thick and long myelinatedfibers in rabbit pupswithout IVH controls (×10 and boxed area in high magnification in ×40). (**c**,**d**): Reduced and sparse of myelinatedfibers with less densityin IVH saline control pups (×10 and boxed area in high magnification in ×40). (**e**,**f**): The ICV USSC injected pups with IVH showed partialrecovery of myelinfiber formation with more number of MBP positive cells (×10 and boxed area in high magnification in ×40). Scale bar, 100 µm.

Different injection methods and reasonable extrapolation mechanisms provide further protection for clinical applications. In terms of injection modalities, a mouse model suggests that MSC intraventricular transplantation significantly attenuates the increase in inflammatory cytokines (IL-1α, IL-1β, IL-6, TNF-α), prevents ventricular hydrocephalus after IVH, and improves sensorimotor function [95]. Another study using a rat model concluded that due to IC, more residual cells in the lateral ventricle over time had the same effect in preventing intraventricular hydronephrosis and sensorimotor impairment and reduced brain damage, such as inflammation, cell death, reactive glial hyperplasia, and delayed myelination after severe IVH, as compared to systemic injection of MSCs [82]. Thus, for objective reasons, such as the operational difficulty of IC and neonatal vulnerability, a less-invasive IV may be a better option in cases of similar efficacy [82]. Different animal models’ construction and extrapolation mechanisms may be subject to significant deviations. Currently, IVH animal models are mainly rats, rabbits, and sheep. Intraventricular injection of maternal blood (for example, in mouse models) extrapolated to the spontaneous rupture of blood vessels in human neonates causes a significant difference in physiological and biochemical changes [96]. Therefore, the IP glycerol-induced intraventricular hemorrhage rabbit model may be closer to the pathophysiology of neonatal IVH. MSCs were found to improve motor and neurological functions, reduce endogenous cell death and microglial infiltration, and increase myelin gene expression in juvenile IVH rabbit models [97] (Figure 8). Therefore, preclinical studies have shown that SC therapy for IVH has a positive significance in terms of mechanisms, administration methods, and animal models. Basic research for IVH based on stem cell therapy is presented in Table 2.

The clinical value of SC treatment is encouraging. A case report from Turkey described an extremely premature infant (27 + 5 weeks of gestation, body weight of 950 g) with grade III intracranial hemorrhage treated with MSCs after the full onset of epilepsy (6 days after birth). At 4 weeks of administration, a normal head ultrasound was reported. At 2 years of follow-up, the patient was generally in good condition with normal neurological examination and development [99]. Human milk contains a large number of neurotrophic factors, such as BDNF, insulin-like growth factor (IGF-1), and HGF [100,101,102], which are beneficial for the long-term behavioral development of children [103]. A retrospective cohort study of very-low-birth-weight infants with 3/4 degree IVH (experimental group, *n* = 16; control group, *n* = 15) found that the incidence of intraventricular perforation defects (21% vs. 58%), progressive ventricular dilatation (71% vs. 91%), and post-IVH hydrocephalus requiring surgical treatment (50% vs. 67%) decreased, indicating that early intranasal breastfeeding may have a beneficial effect on nerve development in premature infants [104]. A phase I single-arm single-center trial in South Korea (*n* = 9, average gestational age 26.1 ± 0.7 weeks, average birth weight 808.85 g) found no intolerance or cytotoxicity in children with severe IVH upon an increase in the dose of UCB-MSCs (the first three received low-dose MSCs, 5 × 10^6^ cells/kg, and the second six received high-dose MSCs, 1.0 × 10^7^ cells/kg) [105]. Treatment is safe and feasible, but more samples from II clinical trials are needed to determine doses, injection methods, evaluation criteria, and prognosis. 

The current clinical treatment cannot promote the regeneration of lost nerves. SC therapy is a promising treatment for reducing mortality and the incidence of complications after IVH in premature infants. ESCs and iPSCs have the potential to differentiate into cells of all organs. However, the regulatory mechanisms of stem cell differentiation, migration, and fusion with surrounding tissues and stem cell termination after transplantation are still unclear, leading to their potential tumorigenicity. More importantly, current research has not yet formally established a true synaptic connection between the transplanted stem cells or their progeny cells and the host neurons. There are only three registered clinical trials on the clinical trial registration website, one of which is the completion of a phase I clinical trial (NCT02274428), as described above. One study on IVH treatment with intranasal breast milk (NCT04225286) and one follow-up study on IVH treatment with MSCs (NCT02673788) were excluded. Further studies are required to evaluate the optimal delivery modality, dose, duration, efficacy, and safety.

### 4.3. Bronchopulmonary Dysplasia

BPD is defined as the need for oxygen support at 28 days after birth and is classified according to the severity of respiratory support at 36 weeks of gestation. At the onset of BPD in children, pulmonary angiogenesis is impaired, and there is a lack of effective alveolar gas exchange [106]. Therefore, the treatment of neonatal BPD needs not only to repair the damage, but also to promote the continued growth of the lungs to meet the growth needs. Therefore, SC therapy may be a promising therapeutic option.

**Figure 9:**
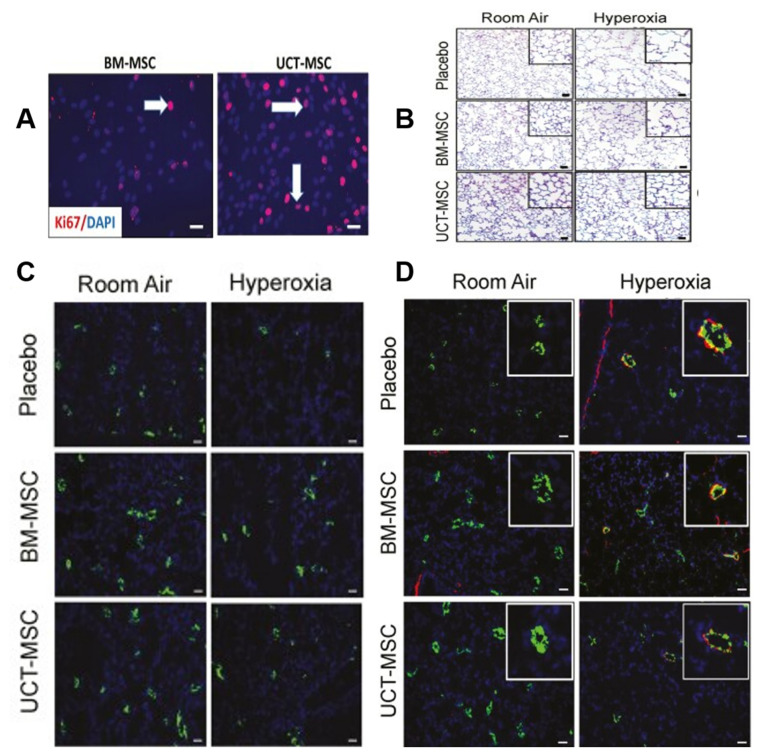
(**A**) Photos taken at 40× magnification show an increase in ki67-positive cells (red signal) after 48 h of UCT-MSC culture compared to BM-MSCs. Arrowheads indicate Ki67-positive cells (red signal). (**B**) Staining lung tissue sections with hematoxylin–eosin showed that BM-MSCs or UCT-MSCs improved alveolar structure in hyperoxia-exposed rats. Original magnification 100×. Scale bar = 50 μm. (**C**) Staining lung sections with vWF antibody showed that BM-MSCs and UCT-MSCs also promoted pulmonary angiogenesis and capillary formation. Original magnification 100×. Scale bar, 50 μm. (**D**) Lung sections stained with von Willebrand factor (green), α-smooth muscle actin (red), and DAPI (blue) showed that both BM-MSCs and UCT-MSCs could improve experimental BPD vascular remodeling. Original magnification 100×. Scale bar, 50 μm [110].

The primary causes of BPD include hyperoxia, inflammation, and oxidative stress [107]. MSCs can inhibit lung inflammation and reduce lung injury, pulmonary hypertension, and pulmonary fibrosis. In a BPD rat model, UC-MSCs reduced apoptosis, calpain I expression, active oxygen production, and abnormal elastin expression and deposition in vitro and promoted proliferation, anoxia-inducing factor-1a expression, VEGF secretion, and human umbilical vein endothelial cell lumen formation; in vivo, they can restore alveolar structure and pulmonary function, improve pulmonary hypertension, and increase vascular density [108,109]. UC-MSCs and BM-MSCs have similar effects on pulmonary angiogenesis and remodeling, reducing pulmonary hypertension; however, UC-MSCs are superior to BM-MSCs in inhibiting pulmonary macrophage infiltration, promoting pulmonary epithelial wound healing, expressing more anti-inflammatory factors (IL-10 and TSG-6), and protecting alveoli [110] (Figure 9). Both human umbilical cord stem cells (hUC-SCs) and hUCB-MSCs prevented and treated BPD, and long-term (6 months) evaluation showed no adverse lung effects from either strategy. The exercise capacity and lung structure continue to improve in rats with BPD [111]. Therefore, UC-MSCs may be the most reliable raw material for preventing and treating BPD in rat models. In addition, in BPD premature rabbit models, the upregulation of VEGF expression in AFSCs enhanced their ability to prevent or reverse lung injury and significantly improved lung parenchyma, vascular structure, and function [112]. Therefore, the extrapolation mechanisms of different models require further exploration.

**Figure 10:**
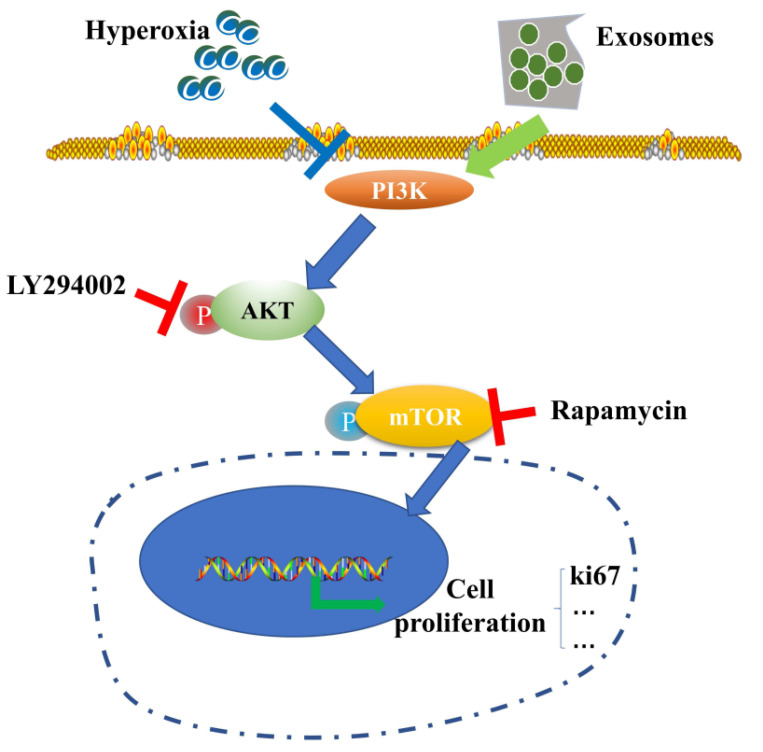
Schematic diagram of BM-MSC-derived exosomes mediating the inhibition of hyperoxia-induced apoptosis by the PI3K/Akt/mTOR/Ki67 pathway under hyperoxia [113].

Acellular therapy is another popular research topic. Yang et al. found that exosomes from BM-MSCs regulate the expression of related proteins through the PI3K/Akt/mTOR signaling pathway, thereby preventing hyperoxia-induced apoptosis of alveolar II epithelial cells [113] (Figure 10). Another study found that exosomes derived from hUCB-MSCs restored alveolar structure and pulmonary function in BPD rats, improved pulmonary hypertension, increased the number of ki-67-positive lung cells, and decreased the number of TONEL-positive lung cells by protecting the PTIAECs (II alveolar epithelial cell markers) and PVECs (VEC markers) associated with the PTEN/AKT signaling pathway [114], and exosomes derived from hUCB-MSCs improved alveolar alveolarization and pulmonary artery remodeling in the injured lung in long-term models [115]. Another study found that extracellular vesicles derived from hADSCs carrying miR-21-5p reduced inflammation and apoptosis, increased cell viability, and reduced oxidative stress in mice with oxygen-induced lung injury [116].

**Figure 11:**
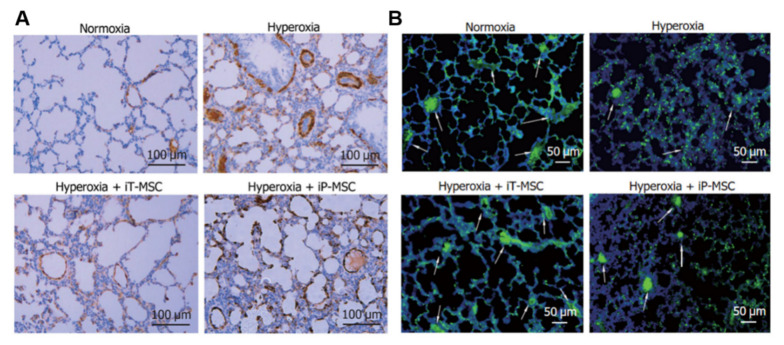
(**A**) To determine the effect on hyperoxia-induced pulmonary vascular remodeling, a study completed smooth muscle actin antibody staining of lung tissue α smooth muscle actin on postnatal day 21 in rats. Scale bar, 100 μm. (**B**) To determine the effect of hyperoxic exposure on the number of peripheral pulmonary blood vessels, lung sections were stained with von Willebrand factor. White arrows highlight stained pulmonary vessels [119]. Scale bar, 50 μm.

**Figure 12:**
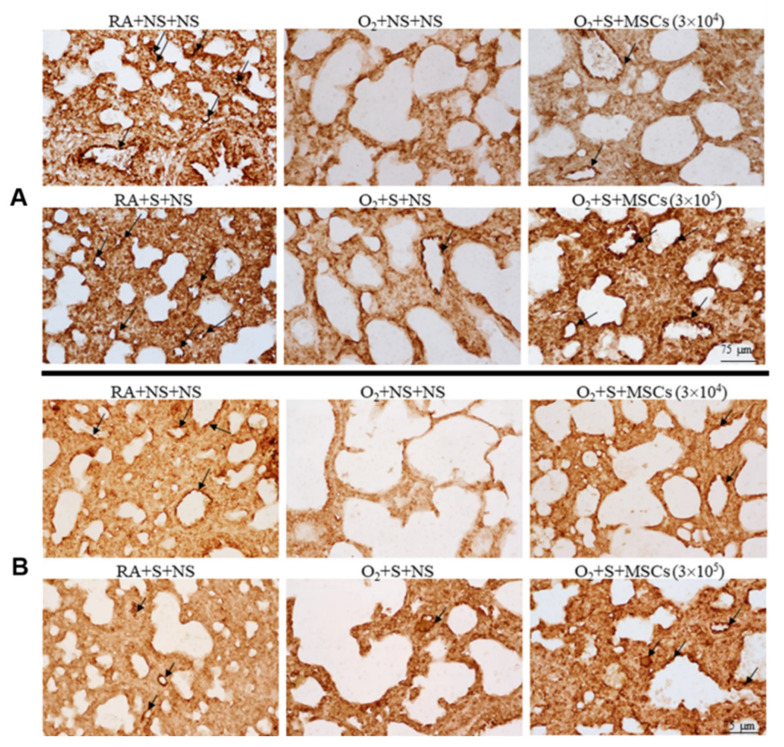
Fourteen-day-old rats are exposed to RA or hyperoxia after birth and treated with NS or surfactant on day 4 and NS or hUC-MSCs (3 × 10^4^ or 3 × 10^5^ cells) on day 5 after birth. (**A**) The figure shows lung sections stained with vWF on postnatal day 14. Surfactant and hUC-MSC (3 × 10^4^ or 3 × 10^5^ cells) treatment significantly increased hyperoxia-induced vascular density decline. The surfactant + hUC − MSC (3 × 10^5^ cells) group had a more significant increase in vascular density than the surfactant + hUC − MSC (3 × 10^4^ cells) group. Vascular endothelium with vWF immunoresponsiveness is indicated by a black arrow. (**B**) The VEGF immune response was detected in vascular endothelial cells. The expression level of VEGF protein in the hyperoxia + saline group was significantly lower than that in the hyperoxia + saline and surfactant group. Black arrows indicate positive staining for vascular endothelial VEGF. MSCs, mesenchymal stem cells; NS, saline; O_2_, oxygen-rich atmosphere; RA, air; S, surfactant; VEGF, vascular endothelial growth factor [120].

More attention should also be paid to injection mode, dosage, and safety. Intratracheal injection of BM-MSCs improves survival and exercise tolerance and reduces alveolar and pulmonary vascular injury, pulmonary hypertension, and inflammation [117]. Intranasal administration of hUCB-MSCs has no significant effect on BPD rats. In contrast, intravenous administration of MSCs can restore damaged lung tissue (lung compliance, elasticity, and pressure–volume ring), and early administration of SCs may reduce or shorten the acute lung injury period [118]. Activation of HO-1 expression and the JAK/STAT signaling pathway by intratracheal injection of hUC-MSCs improves hyperoxia-induced lung, heart, and kidney injury, and the therapeutic benefits of local intratracheal injection and intraperitoneal administration are equivalent [119]. Figure 11 shows whether UC-MSC could salvage hyperoxia-induced peripheral pulmonary vascular loss and peripheral pulmonary artery remodeling. Chou et al. found that two consecutive days of intratracheal administration of alveolar surfactants (hUCB-MSCs) reduced inflammatory factors, increased VEGF expression and vessel density, and improved survival in BPD models. Larger doses of hUCB-MSCs (3 × 10^5^ times) were more effective than smaller doses (3 × 10^4^ times) [120] (Figure 12). Several clinical studies have found that intravenous UCB-MSCs are safe and effective in reducing pulmonary fibrosis [121,122,123]. A single-center, non-randomized controlled trial (experimental group, *n* = 29; control group, *n* = 33) found that IV human mononuclear MSCs prevented moderate to severe BPD in preterm infants and improved long-term neurodevelopmental outcomes [124]. In conclusion, SC treatment is safe, reliable, and has a certain effect on long-term prognosis. Tracheal or intravenous injections were commonly administered. However, the optimal dose requires further investigation. In summary, endogenous stem cells play a key role in lung development. At present, MSC transplantation for BPD is still in the clinical research stage, and its optimal indication and optimal therapeutic time window need to be explored in future clinical trials. In addition, the safety of MSC transplantation is of the greatest concern. In contrast, the treatment of MSC-derived EVs has obvious advantages over MSC transplantation: EVs cannot self-replicate and there is no risk of tumorigenesis. EVs are less immunogenic than parental cells, making allogeneic administration possible. However, the mechanism of MSC-derived EVs in the treatment of BPD is still unclear, and a series of problems such as the quality and quantity of MSC-derived EVs needed to reach clinical level of use need to be solved urgently. Basic research for BPD based on stem cell therapy is shown in Table 3.

These results demonstrate the advantages and great potential of MSC application; however, the method of administration, dosage, and treatment window need to be further clarified. At present, there are 22 trials registered on the website of clinical trials, 7 of which have been completed (NCT02443961, NCT02381366, NCT01297205, NCT01828957, NCT01897987, NCT02023788, and NCT02639676), 5 of which are conducting recruitment (NCT03645525, NCT03631420, NCT04003857, NCT03392467, and NCT04255147), 1 of which is being actively prepared (NCT01632475), one of which is being suspended (NCT03857841), and 8 of which the progress is unknown (NCT04062136, NCT03558334, NCT03601416, NCT03378063, NCT03873506, NCT03683953, NCT03774537, and NCT01207869). The So Yoon Ahn [123] team conducted a phase II double-anonymized, randomized controlled clinical trial (experimental group, *n* = 33; control group, *n* = 33) of premature infants (gestational age 23–28 weeks) who received mechanical ventilator support and whose breathing deteriorated from the 5th to the 14th day after birth; the experimental group received SCs (1 × 10^7^ cells/kg) or placebo. MSCs significantly reduced inflammatory cytokine levels in tracheal aspirates; however, the incidences of death or moderate/severe BPD were similar (52% vs. 55%). In the subgroup analysis, the incidence of severe BPD in MSC transplantation was significantly improved (53% vs. 19%) at 23–24 weeks of gestation compared to that in infants at 25–28 weeks. This study shows that SC treatment is safe and feasible; however, the sample size is small, and larger multicenter prospective randomized controlled trials are needed to explore SC therapy in BPD further.

### 4.4. Necrotizing Enterocolitis

NEC is an acute necrotic intestinal disease caused by many pathogenic factors during the perinatal period and is characterized by abdominal distension, vomiting, and hematochezia. Currently, the main treatments include fasting, gastrointestinal decompression, antibiotics to prevent infection, parenteral nutrition, and surgical resection of the diseased intestinal segments [126,127,128]. However, children with NEC often experience long-term complications, such as short bowel syndrome, stunting, and neurological sequelae [129,130]. Therefore, novel control measures must be developed.

**Figure 13:**
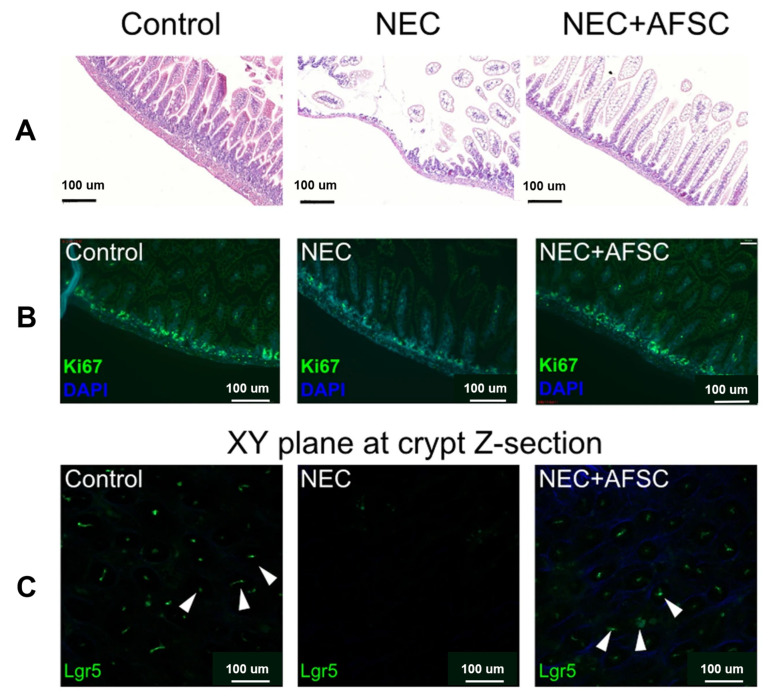
NEC induction was performed 5–9 days after birth, AFSCs were injected intraperitoneally on p6 and p7, and animals were sacrificed on P9. (**A**) Administration of AFSCs attenuated NEC-induced intestinal injury. (**B**) Intestinal epithelial proliferation (Ki67), which was reduced in NEC, was restored after AFSC administration. (**C**) Using 3D reconstructions of ileal tissue, active intestinal stem cells observed in vivo after NEC induction showed a reduction in Lgr5 + ISC; some of these cells are indicated by white arrows and were expressed in NEC and restored after AFSC treatment [137].

**Figure 14:**
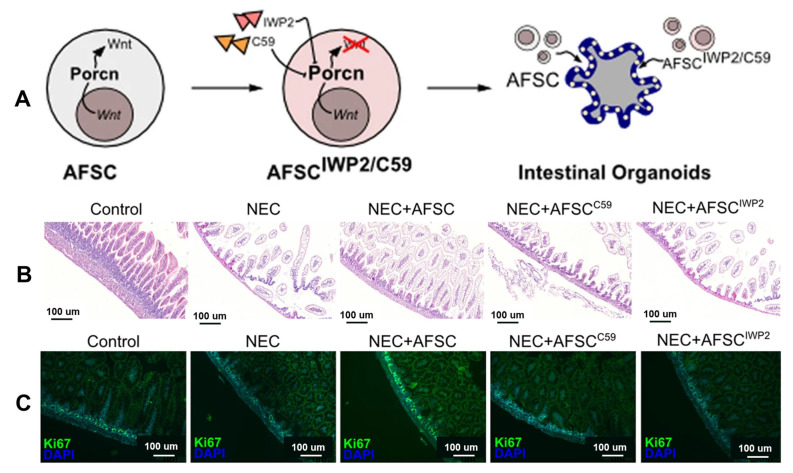
(**A**) AFSCs promote intestinal organoid ISC and epithelial cell proliferation via the Wnt signaling pathway. The study included intestinal organoids with AFSCs treated with two known Porcn inhibitors (Wnt-c59 and IWP2, which are involved in Wnt maturation and release). (**B**) The Wnt signaling pathway plays an important role in reducing intestinal injury in NEC. Ileum sections from NEC mice treated with C59 and IWP2-treated AFSCs showed increased villus damage compared with NEC + AFSCs. (**C**) wnt-AFSC treatment promotes epithelial cell proliferation (Ki67) [137].

The pathogenesis of the disease is unclear, and studies have suggested that premature infants are susceptible to immune responses, vascular damage, and microbial imbalance due to intestinal susceptibility and hyperresponsiveness [131]. Furthermore, three major inflammatory markers, TNF-α, IL-1b, and NFKB, have been linked to NEC [132,133,134]. SCs from different sources have different mechanisms of action in NEC, and their roles in clinical practice must be compared. Studies have shown that bioactive factors in amniotic fluid inhibit TLR4-mediated epithelial injury, reduce inflammatory signals, and promote intestinal maturation in premature infants [49,135]. The prophylactic use of amniotic fluid mesenchymal stem cells (AF-MSCs) can reduce the severity of NEC and mucosal inflammation [136]. Figure 13 shows that AFSCs improved intestinal injury in NEC, restored epithelial regeneration, and increased the number of active intestinal SCs [137]. It can also reduce the incidence of NEC through the COX-2 dependent mechanism and differentially expressed Wnt/βcatenin pathway gene, improve intestinal function, reduce apoptosis and inflammation (Figure 14) [49,137], promote the proliferation of intestinal mucosa and activation of endogenous SCs, and reduce the risk of tumor formation [136]. Intraperitoneal injection of intestinal neural SCs improves intestinal function in NEC mice [33,138]. 

**Figure 15:**
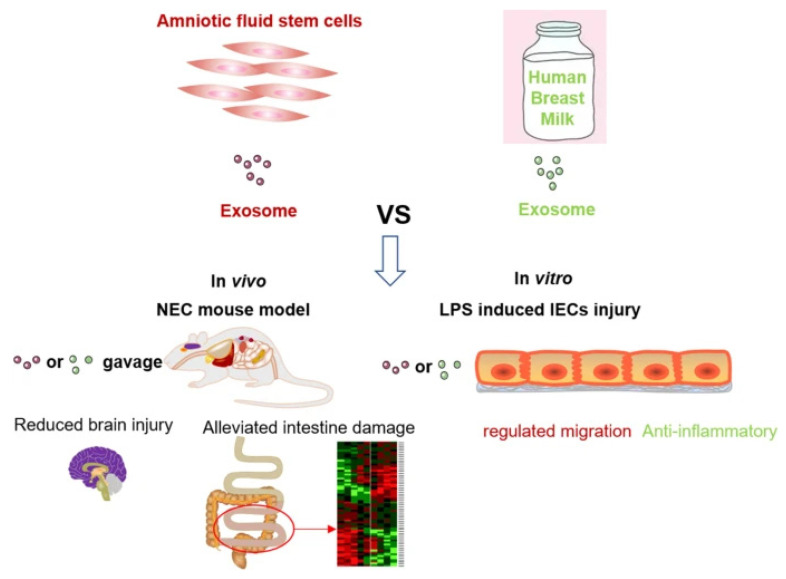
Comparison of the effects of AFSC-exo and HBM-exo interventions in mouse models of NEC. Both exosomes reduce NEC-related intestinal damage, significantly reduce NEC scores, and reduce systemic and ileal inflammation and NEC-related brain damage [140].

SCs and extracellular vesicle therapies have both advantages and disadvantages. McCulloh et al. concluded that exosomes derived from SCs were not significantly different from SCs when treating NEC mice and significantly reduced NEC incidence and severity [138,139]. In vitro, exosomes derived from human breast milk SCs (HBM-exos) preferentially inhibited the inflammatory response of intestinal epithelial cells, whereas exosomes from hAFSCs (AFSC-exos) preferentially regulated the migration of intestinal epithelial cells; in vivo, the number of ileal crypts recovered faster after intervention with HBM-exos (Figure 15) [140]. BM-MSCs produce a variety of soluble mediators, such as cytokines, growth factors, microRNAs, and exosomes, to mitigate NEC-related injury [141]. Li found that AFSC-exos could also reduce NEC intestinal damage by activating the Wnt signaling pathway and increasing cell proliferation. However, exosomes must be administered during NEC induction to prevent intestinal damage. Therefore, AFSC-exo administration is a potential new strategy for treating NEC [142]. In summary, the kind of treatment that can provide better efficacy for children with NEC requires further discussion. Basic research for NEC based on stem cell therapy is presented in Table 4.

The intestinal epithelium is the most important barrier preventing foreign antigens and toxins from entering the systemic circulation [144]. SC therapy not only alleviates the damage caused by the disease itself, but also improves intestinal epithelial absorption and intestinal adaptation [145], which is essential for recovery from short bowel syndrome after NEC. A case report on NEC (involving the whole colon and 80% jejunum and ileum) after intestinal ischemia–reperfusion in full-term newborns (4 days after birth) after supraventricular tachycardia [146] mentioned that the patients were using the UC-MSC growth after surgical excision of the necrotic intestinal segment and found that the nervous system development of the children was similar to that of their peers. However, a recent study did not confirm whether SC transplantation is prophylactic or therapeutic for neonatal NEC and did not determine the treatment window [147]. Ideally, clinicians would offer SCs to children with definitive NEC that have not yet progressed to the stage of surgical intervention, namely BELL IIA, IIB, and IIIA. However, in clinical practice, many children with BELL IIA can recover only through observation and intestinal rest. Therefore, using SCs in children with nonsurgical NEC beyond IIA may be the best choice [148]. Improving the sensitivity of NEC detection may provide guidelines for determining precise SC treatment time windows. These studies provide a new approach to solving problems in regenerative medicine and increase the possibility of improving the prognosis of children with NEC requiring surgical treatment. The migration of SCs to the site of intestinal proliferation and necrosis was seldom observed in the above experiments, and most of the SCs were regulated by a paracrine mechanism. Therefore, studying the paracrine mechanism of SCs is an important research direction, and the injection method and administration dose may be of more significance. To date, only one clinical trial involving umbilical cord blood mononuclear cells for the treatment of digestive system disorders (including NEC) in premature infants (NCT05138276) has been conducted using the clinical trial registration website. Therefore, our summary of the mechanisms and preclinical studies may be helpful for further studies on SC therapy for NEC.

### 4.5. Retinopathy of Prematurity

ROP is an eye disease characterized by abnormalities in the blood vessels of the developing retina. Currently, the main treatment methods include cryotherapy, laser therapy, vitrectomy, and bevacizumab. However, these methods focus on the late stages and do not address the underlying pathological defects. Each has significant safety implications [149], such as ulcerative keratitis, intravitreous vascular hyperplasia, retinal detachment, and sustained retinal vascularization [150,151,152,153]. Therefore, it is important to search for safer and more effective treatments.

Researchers in various countries have explored several therapeutic mechanisms for ROP. VEGF and HGF regulate the migration and proliferation of retinal endothelial cells [154,155]. Intravitreal angiogenesis can be induced by different angiogenic factors, such as VEGF, placental growth factor [156], IGF-1 [157], or erythropoietin [158]. IGF-1 and growth hormones stabilize the retinal vessels and reduce the lethal effects of high oxygen concentrations on endothelial cells [159]. Early angiopoietin 1 (Angular-1) can promote the construction of healthy vascular networks; inhibit abnormal vascular proliferation, vascular leakage, neuronal apoptosis, and neuronal dysfunction in the retina in OIR models; and salvage vascular retinopathy [160]. A clinical study (including 29 preterm infants with ROP at 33 weeks, 29 non-ROP preterm infants at 33 weeks, and 30 healthy term infants) found that the number of endothelial progenitor cells (*p* = 0.03) and concentrations of VEGF (*p* = 0.048), HGF (*p* = 0.001), and SDF-1 (*p* = 0.001) were significantly higher in ROP preterm infants than in non-ROP preterm infants. Increased plasma concentrations of VEGF and HGF are associated with endothelial progenitor cell mobilization; however, a causal relationship is unclear [161]. Based on these findings, SC therapy may be feasible for treating ROP.

**Figure 16:**
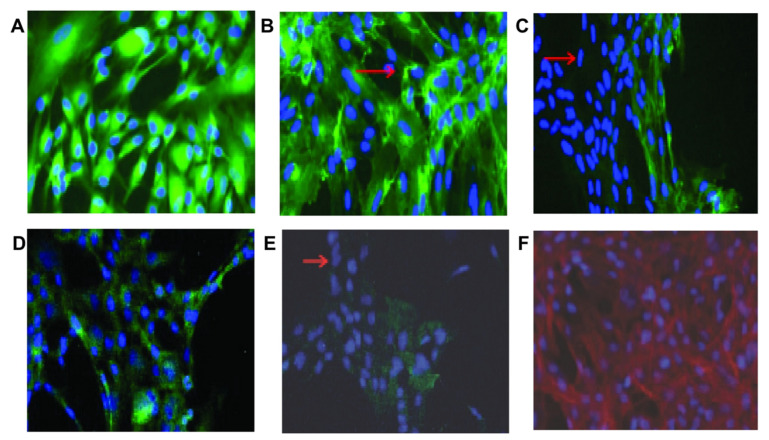
Immunohistochemical staining showed that RSCs expressed proteins associated with different retinal cell types. Almost all RSCs express (**A**) GFAP (astrocytes), (**B**) isolectin (microglia), (**D**) and recoverin (photoreceptor cells); most cells do not express (**C**) PKC (rod bipolar cells) and (**E**) RPE65 (RPE cells). The arrows show cells that do not express the protein under study. (**F**) Staining with rhodamine phalloidin and DAPI shows actin (red) and nuclei (blue) [164]. Scale bar is not mentioned.

SCs used to treat ROP are mainly derived from the cord blood, bone marrow, or peripheral blood. BM-MSC transplantation can promote tissue repair and angiogenesis [162] and increase the expression of VEGF, Ang-1, hematopoietic growth factor, and transforming growth factor-β [163]. Retinal SCs (RSCs) can migrate, differentiate, and integrate into the retina (Figure 16) [164]. The co-culture of BM-MSCs and RSCs transfected with Angular-1 can induce the differentiation of RSCs into β-tubulin and PKC, promote the expression of Ang-1 and IGF-1 and proliferation and differentiation of RSCs, and effectively improve the therapeutic effect of OIR-ROP in rats with retinal injury [165]. Other studies have suggested that BM-MSCs transfected with Ang-1 could improve the morphology of the injured optic nerve and repair the myelin sheath of nerve fibers, demonstrating its preventive and therapeutic effects on optic nerve injury in OIR rats [166]. In addition to BM-MSCs, SCs from other sources also play a role in treating this disease. Kim, Kyung-Sul’s research suggests that amniotic MSCs can migrate to the retina, inhibit neovascularization by secreting high-dose TGF-β, and increase the normal vascularization region of proliferative retinopathy [167]. Hematopoietic SCs injected into newborn mice may participate in the formation of blood vessels during retinal development [168] and may also target activated glial cells to mitigate optic nerve injury [169]. There is no doubt that stem cell transplantation has made good progress in the research on ROP and shows a promising prospect for clinical application, especially as the drawbacks of anti-VEGF drugs continue to emerge. However, the mechanisms of how stem cells reestablish the balance between pro-neovascular growth factors and inhibitory neovascularization factors have not yet been investigated. Currently, there are no trials on the SC treatment of ROP on the clinical trial registration site. Therefore, although the above studies show that SC treatment of ROP is promising, a large number of clinical data are needed to verify the feasibility of its application further. Basic research for ROP based on stem cell therapy is presented in Table 5.

## 5. Challenges and Prospects

With an increase in the neonatal survival rate, the incidence of neonatal diseases is also increasing, and the limitations of existing treatment methods are gradually appearing. New control methods are urgently needed to achieve a better prognosis. SC therapy has become a new research focus owing to its variability and versatility. However, there are many challenges in the use of SCs in preclinical therapy. Many countries have approved dozens of MSCs for the treatment of adult diseases. Mature protocols for SC therapy are available for adults, but research on newborn populations is lagging, and a great number of data are needed to advance the research design. There are few basic studies on the long-term prognosis of SC therapy, most focusing on transient changes in tissue and function. Simultaneously, the indications, treatment time window, injection method (route and dose), and long-term benefit monitoring of SC treatment have not been unified and require further studies. 

### 5.1. Preparation of SCs

Access to SCs comes from various sources, and proliferative capacity and senescence variables are inconsistent in different batches [171]. Therefore, there are many challenges in determining the mass production and quality of SCs for large-scale clinical applications. BM-MSCs, AF-MSCs, UC-MSCs, and placenta-derived mesenchymal stem cells (PMSCs) have the advantages of low immunogenicity and immunity. They can be transplanted by autotransplantation and allogeneic donors into NEC infants [13]. However, the number of BM-MSCs is limited, and multiple generations are required to reduce the presence of hematopoietic precursor cells in the sample. Obtaining multiple generations requires a high level of experimental technique and, therefore, has a narrow range of applications [172,173]. AF-MSCs can be collected during routine amniocentesis and cesarean section, grow stably in culture medium [138,174,175], and are widely used. Considering the different disease mechanisms and stem cell sources, suitable SCs must be selected individually. Currently, systematic evaluation systems are lacking.

The prompt amplification of a sufficient number of effective SCs remains to be explored. How are effective SCs defined? SCs have high proliferation capacity and multi-directional differentiation ability [176]. For example, cells expanded by fibroblast colony-forming units produce bone tissue in nude mice [177]; however, only a portion of these cells are polyclonal [176]. The minimum criteria for MSCs identified by the International Society for Cell Therapy are as follows: (1) under standard culture conditions, MSCs must adhere to plastic substrates; (2) CD105-, CD73-, and CD90-positive and CD45-, CD34-, CD24-, CD11b-, CD79a- or CD19-, and HLA-DR-negative; and (3) under in vitro standard differentiation conditions, MSCs can differentiate into osteoblasts, fat cells, and chondrocytes. Therefore, most publications refer to fibroblast-like plastic adherent cells as MSCs; however, many do not meet the basic characteristics of SCs [176]. Therefore, it is not appropriate to classify this class of MSCs as SCs. Therefore, clarifying how to extract truly effective MSCs may be a promising approach for cell therapy. Most studies have concluded that introducing specific genes into the target cell genome is safe while maintaining the MSC differentiation capability and expression of the same markers as their non-immortalized counterparts. For example, transfection through HPV16 E6/E7 gene transduction enables the immortalization of human MSCs and reduces the risk of tumorigenicity [178]. In contrast, co-transfection of hTERT/SV40 (hADSCs-TS) or hTERT/HPV E6/E7 (hADSCs-TE) can immortalize hADSCs (which can be cultured for up to 1 year with the cell population doubling level reaching 100) and retain the ability to secrete large amounts of HGF and VEGF [179]. MSCs with TERT can overcome the restriction of primary MSCs replication and prolong their longevity by controlling the expression of key enzymes of extracellular nucleotides/nucleotides, for example by in vitro changes in the purine metabolism of MSCs, especially the adenosine pathway [180]. These studies may alter the efficacy of MSCs in tissue engineering for regenerative medicine. A sufficient number of SCs is a prerequisite for successful treatment.

### 5.2. Application of Exosomes from SCs

In terms of mechanism, extracellular vesicles derived from SCs do not induce immunogenicity responses similar to those of SCs and therefore do not have tumorigenic effects that might be present in SC therapy, which may be of special value in neonatal therapy. In terms of size, extracellular vesicles are smaller than SCs, allowing them to cross the blood–brain barrier and treat some animal models of traumatic brain injury [181,182,183]. However, the half-life and activity of extracellular vesicles in vivo are unknown. Future studies on the content of extracellular vesicles may shed light on the mechanisms by which specific messengers participate in SC therapy and provide excellent answers for applications in regenerative medicine. The effective excitation range of extracellular vesicles derived from MSCs administered via different routes and the potential systemic effect are uncertain in clinical practice. Standardization of MSCs quantities remains controversial [114].

### 5.3. Clinical Application

Studies have shown that intravenously injected SCs can form cell masses in the lungs, possibly with sarcomatous lesions of the lungs in mice [184,185]. Multiple pulmonary embolisms and infarctions are caused by multiple intravenous infusions of autologous adipose-tissue-derived cells [84]. Therefore, routine intravenous drug delivery is risky in SC therapy. Therefore, it is necessary to develop a more suitable method of administration to treat these diseases. The administration methods for different diseases are different, and the dosage and treatment course must be further explored.

Treatment with SCs has potential oncogenic effects and other unknown side effects; however, the prevention and treatment of these effects are not clear. A meta-analysis of 36 prospective clinical trials [186] (*n* = 1012) concluded that treatment with MSCs is safe and not associated with organ system complications, infection, death, or malignancy (*p* > 0.05) but is significantly associated with transient fever (OR 16.82, 95%CI 5.33–53.10). However, the study targeted adults and children with ischemic stroke, Crohn’s disease, cardiomyopathy, myocardial infarction, and graft-versus-host disease and lacked data on newborn populations. Other studies have suggested that MSCs have a potential tumorigenic risk [187,188,189] during treatment; however, the more differentiated the SCs, the lower the risk of this complication. Therefore, SCs with rapid growth and weak self-differentiation ability, such as BM-MSCs and AF-MSCs, should be selected [20,175,186,190]. Currently, the treatment of acellular extracellular vesicles is a promising research direction. In addition, SCs were reviewed by an ethics committee, and informed consent was obtained before clinical trials were conducted to treat neonatal diseases. Therefore, to obtain an optimal solution for the clinical application of SC therapy, there is a need to select the appropriate cells, overcome the difficulties of experimental technology, and implement the practice in every link of clinical application.

### 5.4. Application Prospects of SCs and Tissue Regeneration

Stem cells, such as ESCs, MSCs, and iPSCs, are powerful tools in regenerative medicine. They can differentiate into desired cells and secrete active ingredients under different stimuli, making them a natural choice for cell therapy [191]. Biomaterials play an important role in tissue engineering and regenerative medicine, and their diversity and pluripotency have contributed to the flourishing of this field [192,193]. The combination of SCs and biological materials has been widely used in adults but is less widely used in the neonatal population and needs further exploration. Some studies suggested that the clinical application of SCs is hindered mainly by poorly targeted therapy and inflammation-mediated transplant rejection, and the efficacy of SCs is uncertain [194]. The advent of microneedles has enabled the achievement of targeted therapeutic effects via transdermal administration [191]. Biomaterials can also act as scaffolds, provide a variety of nutritional factors, and induce targeted differentiation of stem cells [192,195,196,197,198], which not only protects cells from reaching targeted treatment sites but also increases the amount of retention after injection, acts as an immune-protective barrier, and creates a microenvironment suitable for cell life [199]. For example, in patients with congenital heart disease, biomaterials are used as cell adhesion platforms and carriers of various regulatory factors, such as growth factors and peptides, providing a microenvironment with sufficient space for cell migration, growth, and differentiation in scaffolds before implantation [200]. Winter et al. designed transplantable tubular hydrogel–collagen micropillars that mimic glial tubes and guide neural progenitor cells to form new neurons to replace damaged or dysfunctional neural tissues [201]. Adding pancreatic progenitor cells to biomaterials to construct an immunosuppression-free transplant in islets may be a treatment for neonatal diabetes [192]. In addition, stem cell therapy requires many SCs, and transporting and preserving them is another challenge for cell therapy. For example, AlgTubes proposed a simple mode of hydrogel transport but did not evaluate its preservation or storage capacity [192]. Biomaterials have great potential in manufacturing stem cells and expanding their applications.

## 6. Conclusions

In summary, SCs have great prospects in treating refractory neonatal diseases and improving prognosis owing to their unique advantages, especially their ability to fight inflammatory and anti-apoptotic effects, reduce oxidative stress, and promote regeneration. This article reviews SCs’ preclinical and clinical applications in refractory neonatal diseases, from traditional to emerging strategies. Several basic and clinical studies have confirmed their safety, feasibility, and efficacy. This article focuses on the current research progress in stem cell therapies, such as those for BPD, IVH, HIE, NEC, and ROP. Various SCs have been found to have beneficial effects on these diseases. Despite progress in this field, finding suitable SCs; preparation; and determining indications, dosage, and ethics remain major challenges. With new technologies and research advancements, stem cell therapy is expected to achieve significant results in treating refractory diseases in newborns.

## Figures and Tables

**Table 1:** Basic research for HIE based on stem cell therapy.

Year	Type of Stem Cells	Injection Method	Subject	Operation	Achievements	Reference
2022	hPSC-EMSCs, hUC-MSCs	Intracranial injection or intranasal delivery	SD rat pups (14 to 16 g) at P7	After ligation of the right common carotid artery, the rats were free to move for 1 h and then exposed to an environment containing 8% oxygen and 92% nitrogen for 2 h	hPSC-EMSCs exhibited higher neuroprotective potential than hUC-MSCs, with a more significant reduction in lesion size and apoptosis in the rat brain after hypoxia–ischemia.	Huang et al. [64]
2021	hADSCs	ih	Male Sprague–Dawley rats (200–350 g)	The right common carotid artery was occluded, lactation occurred for 2 h 30 min after surgery, and then the artery was exposed in a 37 °C water bath to 8% O_2_/92% nitrogen for 90 min	The delayed post-treatment with adult-derived AD-MSCs increased the absolute number of medium-spiny neurons in the striatum after perinatal HI-induced brain injury.	Basham et al. [73]
2019	UC-MSCs, umbilical cord blood mononuclear cells	ic	Sprague–Dawley rats at P7	The left common carotid artery was ligated, followed by free movement for 4–8 h	Transplantation of UC-MSCs or CB-MNCs after HI reduced astrocyte proliferation, prevented striatal neuronal loss, and significantly improved functional brain outcomes after a 28-day recovery period.	Zhang et al. [69]
2019	UCB-MSCs	ic	Wistar rats at P7	The left common carotid artery was ligated and exposed to an environment containing 8% oxygen and 92% nitrogen for 2 h	UC-MSCs exerted neuroprotective effects against hypoxic–ischemic injury by inhibiting apoptosis.	Li et al. [77]
2018	hAFSCs	Intranasal injection	C57BL/6 male mice at P9	The right common carotid artery was ligated and exposed to an environment containing 8% oxygen and 92% nitrogen for 30 min	The hAFSCs exhibited neurorestorative effects in the chronic phase of neonatal hypoxic–ischemic encephalopathy in mice.	Otani et al. [67]
2018	AFMSCs, source of embryo MSCs, ES-MSCs	ic	C57/BI6 mice at P7	After ligation of the left common carotid artery, the rats were free for 2 h and then exposed to an environment containing 8% oxygen and 92% nitrogen for an additional hour	Embryonic stem-cell-derived MSCs have a superior neuroprotective capacity over fetal MSCs in the hypoxic–ischemic mouse brain.	Hawkins et al. [62]
2018	BM-MSCs	ic	Sprague–Dawley at P7	After ligation of the right common carotid artery, the rats were free to move for 1 h and then exposed to an environment containing 8% oxygen and 92% nitrogen for 2 h	Co-culture/transplantation of MSCs enhanced autophagy and neuroprotection by increasing BDNF expression and thus reducing activation of the mTOR pathway.	Zheng et al. [66]
2018	UCB-SCs	Intraperitoneal injection	Wistar/ST pups at P7	After ligation of the left carotid artery, the rats were free for 1 h, then exposed to 8% oxygen for 1 h and free for 6 h	Administration of umbilical cord blood cells temporarily reduces hypoxic–ischemic brain damage in neonatal rats.	Hattori et al. [58]
2017	hUCB-SCs	iv (femoral vein)	CB-17 SCID mice were immunodeficient and at P8	After ligation of the left common carotid artery, the rats were free for 1 h and then exposed to an environment containing 8% oxygen and 92% nitrogen for an additional 30 min	The hematopoietic stem cells/endothelial progenitor cells significantly improved reduced cerebral blood flow in the ischemic penumbra band of newborn mice with HIE.	Ohshima et al. [63]

P: postnatal day; iv: intravenous injection; ic: intracerebroventricular injection; ih: hypodermic injection; BM-MSCs: bone-marrow-derived mesenchymal stem cells; hUCB-MSCs: human umbilical cord blood-derived MSCs; AFSCs: amniotic-fluid-derived stem cells; hAFMSCs: human amniotic fluid mesenchymal stem cells; hADSCs: human adipose-derived mesenchymal stem cells; hUC-MSCs: human umbilical-cord-derived mesenchymal stem cells.

**Table 2:** Basic research for IVH based on stem cell therapy.

Year	Type of Stem Cells	Injection Method	Subject	Operation	Achievements	Reference
2022	Thrombin-preconditioned MSCs	Injected at very low rate (10 µL/min) into the right ventricle	Newborn Sprague–Dawley rats	300 µL of fresh whole blood obtained from the tail vein of the female rat was injected into both lateral ventricles	Thrombin pretreatment significantly increased the therapeutic potential of neonatal rats with induced severe IVH compared to first-time recipients of MSC transplantation.	Jung et al. [98]
2021	hUCB-MSCs	ic	Male neonatal Sprague–Dawley rats	On day 4 after birth, 200 µL of fresh maternal whole blood was injected into the lateral ventricle	Stem cells restored thalamocortical plasticity and rescued cognitive deficits in newborns with intraventricular hemorrhage.	Ahn et al. [92]
2020	hUCB-MSCs	ic	Sprague–Dawley rats	On day 4 after birth, 200 µL of fresh maternal whole blood was injected into the lateral ventricle	BDNF secreted by MSCs protected against brain damage caused by severe IVH.	Ahn et al. [94]
2019	hUCB-SCs	ic/iv	Timed pregnant New Zealand white rabbits	Newborn rabbits (3 days premature) were intraperitoneally injected with 6.5 g/kg glycerin water 3–4 h after birth	Unrestricted adult stem cells (USSCs) derived from human umbilical cord blood were reparative in rabbits with brain and spinal cord injury.	Vinukonda et al. [97]
2018	hUCB-MSCs	ic	Sprague–Dawley rat pups	Four days after birth, 200 µL of fresh maternal whole blood was injected into the lateral ventricle	hUCB-MSC treatment attenuated neuronal loss, promoted neurogenesis, and improved neurocognitive function in IVH-injured neonatal rats through BDNF-TrkB-CREB signaling axis activation.	Ko et al. [93]

iv: intravenous injection; ic: intracerebroventricular injection; hUCB-MSCs: human umbilical cord blood-derived MSCs.

**Table 3:** Basic research for BPD based on stem cell therapy.

Year	Type of Stem Cells	Injection Method	Subject	Operation	Achievements	Reference
2022	hUC-MSCs	Intratracheal injection	Sprague–Dawley rats aged 6–8 weeks	The rats were exposed to 80% FiO_2_ from day 1 to day 14 after birth.	In vivo, HPMSCs restored alveolar structure and lung function, ameliorated pulmonary hypertension, and increased vascular density in a rat model of BPD.	Hao et al. [108]
2021	hUCB-MSCs	Intratracheal injection	Sprague–Dawley rats within 12 h after birth	The rats were exposed to 85% FiO_2_ from day 1 to day 14 after birth.	Daily continuous administration of intratracheal surfactant and human umbilical cord mesenchymal stem cells attenuates hyperoxia-induced lung injury in neonatal rats.	Chou et al. [120]
2021	Exosomes of hUC-MSCs	Intratracheal injection	Sprague–Dawley rats	The rats were exposed to 60% FiO_2_ from day 1 to day 14 after birth.	Intratracheal administration of MSC-derived exosomes improved impaired alveolarization and pulmonary artery remodeling.	Porzionato et al. [115]
2020	hUC-MSCs, BM-MSCs	Intratracheal injection	Sprague–Dawley rats within 24 h of birth	From day 1 to day 21, animals were exposed to 85–90% FiO_2_ in the environment, with short interruptions (<10 min) in animal care each day.	BM-MSCs and UCT-MSCs exhibited significant lung regenerative effects in BPD, but UCT-MSCs inhibited lung macrophage infiltration and promoted lung epithelial cell healing to a greater extent.	Benny et al. [110]
2020	hUCB-MSCs, extracellular vesicles	Intratracheal injection	Sprague–Dawley rats	They were exposed to 85% FiO_2_ from day 1 to day 14 after birth.	hucMSC-sEV restored alveolar structure and lung function and ameliorated pulmonary hypertension in a rat model of BPD.	You et al. [114]
2018	hUC-MSCs	Intratracheal injection	Sprague–Dawley rats	The rats were exposed to 80% FiO_2_ from day 1 to day 14 after birth.	UC-MSC ameliorated abnormal elastin expression in the lungs of hyperoxia-induced BPD models.	Hou et al. [109]
2018	AFSCs	ip	Time-mated pregnant rabbits	They were exposed to >95% FiO_2_ in the environment from day 0 to day 7 after birth.	Upregulation of VEGF expression in hAF-SC showed enhanced potential in preventing/reversing lung injury in preterm rabbits.	Jiménez et al. [112]
2017	BM-MSCs	Intratracheal injection	Sprague–Dawley rats	The rats were exposed to 85% FiO_2_ from day 1 to day 21 after birth.	MSCs secreted SDF-1 to regulate angiogenesis and recruit stem cells, thus attenuating lung injury in a bronchopulmonary dysplasia model.	Reiter et al. [125]

ip: intraperitoneal injection; BM-MSCs: bone-marrow-derived mesenchymal stem cells; hUCB-MSCs: human umbilical cord blood-derived MSCs; AFSCs: amniotic-fluid-derived stem cells; hUC-MSCs: human umbilical-cord-derived mesenchymal stem cells.

**Table 4:** Basic research for NEC based on stem cell therapy.

Year	Type of Stem Cells	Injection Method	Subject	Operation	Achievements	Reference
2022	hAFMSCs or hBM-MSCs	NM	C57BL/6 mice at P6-10	q3h was fed high-calorie and low-lactose formula by tube feeding method. q12h uses 100% nitrogen for 60 s for asphyxiation to achieve stress, and after another 5 min, it is placed in the refrigerator at 4 °C for 5 min for cold stress.	AFSC-exos and HBM-exos reduce the severity of experimental NEC and intestinal injury by different mechanisms.	Hu et al. [140]
2021	Human placenta-derived stem cells	ip	Sprague–Dawley rats	Tube feeding, 4 times a day, hypertonic formula (15 g OH combined with 75 mL IL), LPS (4 mg/g 3 times within 30 h of birth), hypoxia (5% oxygen for 10 min, 3 times a day).	hPSCs showed significant restorative effects on villus–crypt morphology and epithelium.	Weis et al. [143]
2021	AFSCs or BM-MSCs	ip	C57BL/6 mice at P5	Hypoxia exposure and tube feeding of formula and oral lipopolysaccharide (4 mg/kg) for 4 days.	AFSC transiently enhanced the growth of healthy intestinal epithelial cells and prevented the development of experimental NEC.	Li et al. [136]
2018	Exosomes of AF-MSCs, BM-MSCs, AF-NSCs, E-NSCs	ip	Sprague–Dawley rat pups born prematurely at 21 days gestation	Injection of reagents 1 h after delivery; for 96 h, high-calorie feeding (through the oral and gastric routes, formula and lipopolysaccharide) was performed every 4 h, and exposure to hypoxia (90 s in a closed container, ambient oxygen concentration was only 1.5%) and low temperature (10 min at 4 °C) was performed every 8 h.	Stem-cell-derived exosomes are as effective as their stem-cell counterparts in reducing the incidence and severity of experimental NEC.	McCulloh et al. [139]
2017	AF-MSCs, BM-MSCs, AF-NSCs, E-NSCs	ip	Timed-pregnant Lewis rats	Hypercaloric feeding every 4 h after birth, exposure to hypoxia and hypothermia every 8 h, and death at 96 h after birth or at the onset of signs of NEC.	Different types of stem cells (AF-MSCs, BM-MSCs, AF-MSCs, or E-MSCs) could significantly reduce the incidence and severity of NEC to an equivalent degree.	McCulloh et al. [138].

NM: not mentioned; P: postnatal day; ip: intraperitoneal injection; BM-MSCs: bone-marrow-derived mesenchymal stem cells; AFSCs: amniotic-fluid-derived stem cells; hAFMSCs: human amniotic fluid mesenchymal stem cells.

**Table 5:** Basic research for ROP based on stem cell therapy.

Year	Type of Stem Cells	Injection Method	Subject	Operation	Achievements	Reference
2021	MSCs, exosome-MSCs	Intravitreal injection	Mice at P17	Postnatal day 7 (P7) pups were placed in a hyperoxic environment set at 75% O_2_ until P12 to trigger vaso-obliteration. The animals were then returned to room air, and hypoxia-driven neovascularization peaked at P17.	MCSs promoted retinal vascular repair by regulating Sema3E and IL-17A in a model of ischemic retinopathy.	Noueihed et al. [170]
2019	BM-MSCs, RSCs	Intraocular injection	Kunming mice on P3	They were exposed to air for 7 days after birth, then exposed to 80% ± 5% oxygen for 5 days, and finally reexposed to air for 5 days.	Co-culture of Ang-1-BMSCs and RSCs promotes the proliferation and differentiation of RSCs and improves the therapeutic effect of damaged retinal tissue in OIR-ROP mice.	Ma et al. [165]
2018	BM-MSCs	Intraocular injection	Kunming mice at P7	They were exposed to air for 7 days after birth, then exposed to 80% ± 5% oxygen for 5 days, and finally reexposed to air for 5 days.	Ang-1-modified BMSCs may have preventive and therapeutic effects on hyperoxia-induced optic nerve injury in neonatal mice.	Liu et al. [166]
2016	hAMSCs	Intraperitoneal injection	C57BL/6J mice at P7	After birth, the rats were exposed to air, then exposed to 75% oxygen for 5 days, and then returned to air again.	Intraperitoneally injected AMSCs migrated to the retina and inhibited excessive neovascularization in the vascular system through TGF-b1 expression.	Kim et al. [167]

P: postnatal day; BM-MSCs: bone marrow-derived mesenchymal stem cells; MSCs: mesenchymal stem cells; RSCs: retina stem cells; hAMSCs: human amniotic mesenchymal stem cells.

## Data Availability

Not applicable.
